# Effects of resistance training and nutritional support on osteosarcopenia in older, community-dwelling postmenopausal Korean females (ERTO-K study): a study protocol

**DOI:** 10.1186/s12877-024-04667-1

**Published:** 2024-01-17

**Authors:** Byung Chan Lee, Kyung Il Kim, Kang Hee Cho, Chang-Won Moon

**Affiliations:** 1 Department of Physical Medicine and Rehabilitation, Chung-Ang University Hospital, Seoul, Korea; 2 Department of Rehabilitation Medicine, Chungnam National University College of Medicine, 266 Munhwa-ro, Jung-gu, Daejeon, 35015 Korea; 3 Department of Biomedical Institute, Chungnam National University, Daejeon, Korea

**Keywords:** Osteoporosis, Sarcopenia, Osteosarcopenia, Resistance exercise, Protein, Study protocol

## Abstract

**Background:**

Osteosarcopenia is geriatric syndrome defined as the concomitant occurrence of osteopenia/osteoporosis, and sarcopenia. Osteosarcopenia is a relatively new concept in geriatric medicine; however, it may increase the risk of fragility fractures, several morbidities and mortalities, and socioeconomic costs. Although resistance exercises and nutritional support—including protein, calcium, and vitamin D—are potential non-pharmacological management procedures, evidence is still lacking. The objective of this study was therefore to evaluate the effect of combined resistance exercise and nutritional support on the quality and quantity of bone and muscle in postmenopausal females with osteosarcopenia.

**Methods:**

This research proposal presents the protocol for a prospective, single-center, single-blinded, two-armed randomized controlled trial. Thirty-four participants with osteosarcopenia will be recruited and randomly divided into intervention and control groups; both groups will receive nutritional supplements (protein, 40 g; vitamin D, 1600 IU; calcium, 600 mg) daily. The intervention group will undergo 24 weeks of resistance exercise of increasing intensity, achieved through a three-phase step-up process. The primary outcomes will be the changes in skeletal muscle index and bone marrow density of the lumbar spine and femoral neck between the baseline and end of intervention (24 weeks). The secondary outcomes will be the body composition, whole body phase angle, physical function assessment, quality of life, psychological assessment, and bone turnover markers of participants, surveyed at multiple time points.

**Discussion:**

This randomized controlled trial may reveal the effect of resistance exercise and nutritional support on older postmenopausal women with osteosarcopenia. The results will provide evidence for developing proper non-pharmacological management guidelines for postmenopausal women.

**Trial registration:**

Clinical Research Information Service of Republic of Korea, KCT0008291, Registered on 16 March 2023, https://cris.nih.go.kr/cris/search/detailSearch.do/25262.

**Supplementary Information:**

The online version contains supplementary material available at 10.1186/s12877-024-04667-1.

## Background

Osteosarcopenia (OS) is a geriatric syndrome characterized by the loss of bone and muscle tissue [1]. Osteopenia or osteoporosis is defined as low bone quality and bone marrow density (BMD) measured by dual-energy X-ray absorptiometry (DXA) or quantitative computed tomography, with T-scores <-1.0 and -2.5, respectively [2]. Sarcopenia (SP) is defined as a skeletal muscle disease that presents with muscle mass loss, muscle weakness, and functional deficits [3]. With the aging population, the co-occurrence of both conditions (OS) is predicted to increase in the future; moreover, OS may increase patients’ risk of fragility fractures, several morbidities and mortalities, and socioeconomic costs [4–6].

In the past, these two conditions were treated as separate entities; however, muscle and bone are interconnected and share mechanical effects. Growth of the bone and muscle are also interactively coordinated by complex paracrine and endocrine signals [7]; therefore, the combination of these two geriatric conditions is not rare, and simultaneous treatment of both conditions is sometimes considered by geriatric physicians. In the last decade, several non-pharmacological treatment guidelines for patients with osteopenia or osteoporosis [8, 9] and SP [10–12] have been announced and utilized in clinical practice. However, neither pharmacological nor non-pharmacological treatment guidelines have been announced for OS. Understanding the pathophysiology of OS, and performing high quality associated interventions is mandatory prior to developing treatment guidelines.

Sedentary lifestyles and poor nutritional status—including protein, vitamin D, and calcium—are known to be key risk factors for OS [13]. Controlling these risk factors may be key to maintaining the quality of bone and muscle tissue in geriatric patients, and perhaps even reverse the pathologic conditions causing these catastrophic results. The FrOST Study (Franconian Osteopenia and Sarcopenia Trial) aimed to evaluate the effect of high-intensity resistance exercise (HI-RT) and protein supplementation on the quantity of bone and muscle in male patients with OS [14]. The trial reported the effects of HI-RT supplemented with protein (1.5 g/kg/day in the HI-RT group and 1.2 g/kg/day in the control group) and vitamin D (800 IU/day), demonstrating improvements in the Z-score for SP (standardized mean difference [SMD]: 1.40), BMD at the lumbar spine (SMD: 0.72) and total hip (SMD: 0.72), and functional parameters (hand grip strength and gait speed) after 18 months of the intervention [15]. Moreover, they demonstrated the safety of the interventions; most participants completed the intervention program, excluding one male who reported a short period of worsening arthritic pain. Due to the COVID-19 lockdown period, the same authors also evaluated the detraining effects of the same study population for approximately 6 months, finding that the intervention group lost approximately one-third of benefits gained from the intervention, and exhibited higher detraining effects than the control group [16]. These trials suggest that a combination of exercise and nutritional support could prevent a further decline in bone and muscle health, and even reverse the pathological conditions; however, continuous management may be the key to managing these patients.

Our study aimed to determine the effects of multidisciplinary interventions—including resistance exercise and nutritional support (protein, vitamin D, and calcium)—to halt or reverse the decremental aging process affecting the quantity and quality of bone and muscle in postmenopausal females with OS.

## Methods/design

### Overview of research design

**Fig. 1:**
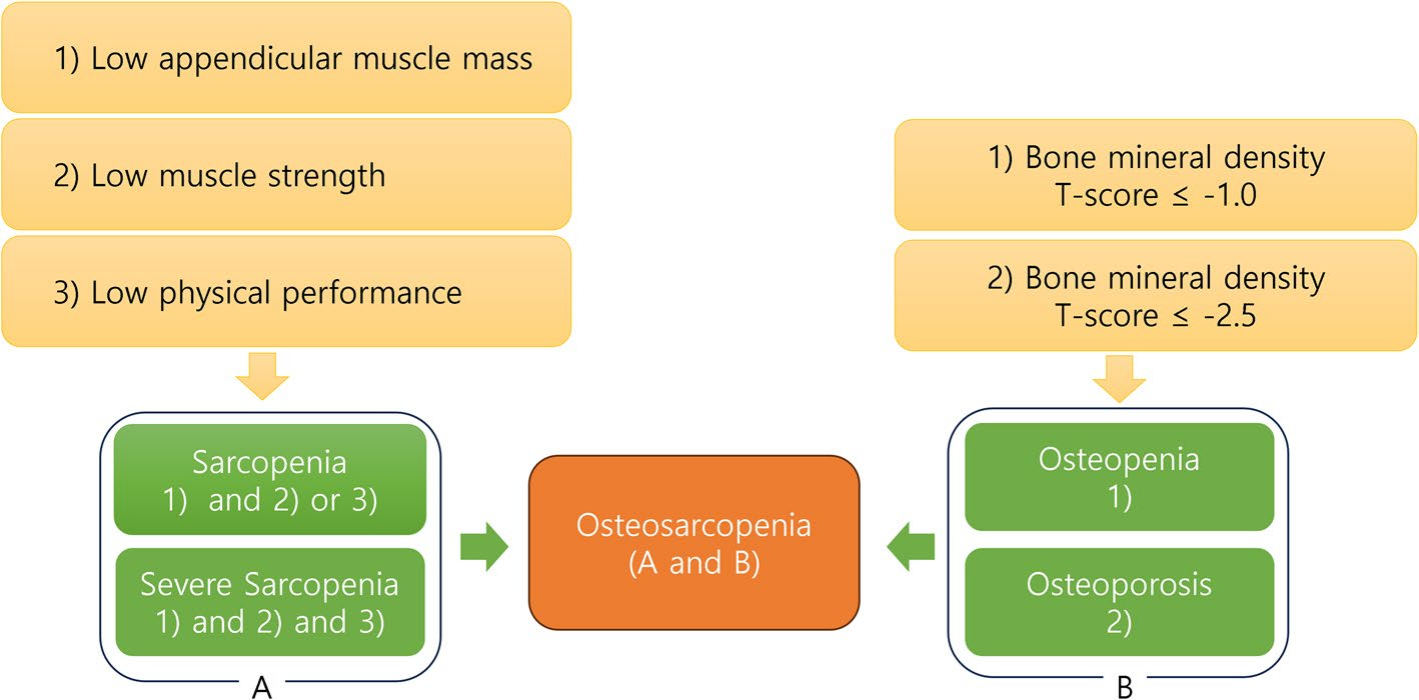
Trial flow diagram

This randomized controlled trial is a double-arm, assessor-blinded trial comparing the effects of resistance exercises combined with nutritional supplementation in postmenopausal females with OS aged ≥65 years in a tertiary urban medical hospital. We followed the SPIRIT guidelines [17], detailed in Supplemental Material 1. The CONSORT flow diagram of the study is provided in Fig. 1.

All patients will provide written informed consent, and the Chungnam National University Hospital's Institutional Review Board has approved the trial's ethical validity (CNUH 2022-12-026). The clinical trial is registered under the Clinical Research Information Service of Republic of Korea (KCT0008291).

### Participants

#### Eligibility criteria

**Fig. 2:**
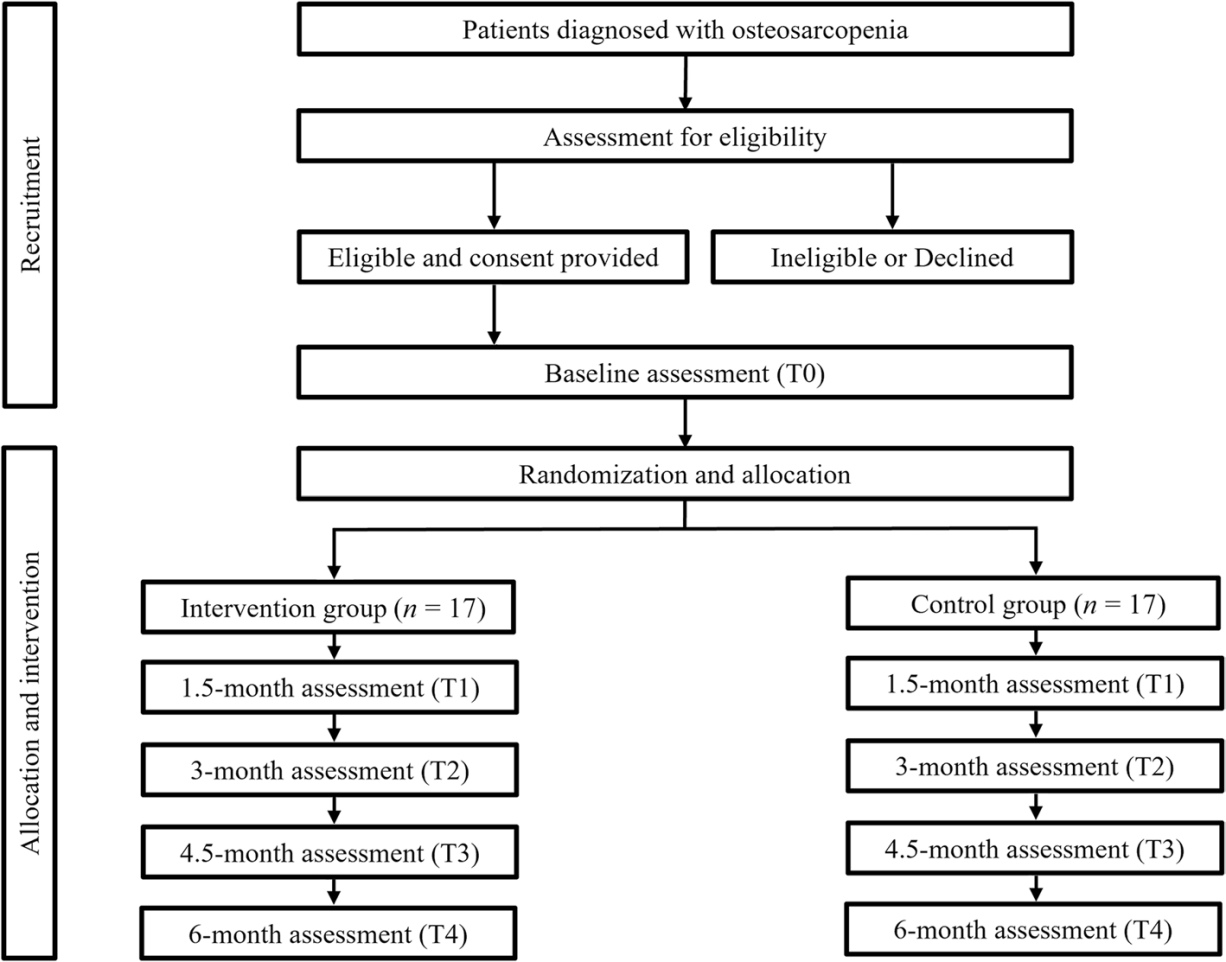
Diagnosis of osteosarcopenia. Patients with sarcopenia or low bone mineral density (osteopenia or osteoporosis) categorized as having osteosarcopenia. The authors utilized diagnostic criteria for sarcopenia as outlined by the Asian Working Group of Sarcopenia.; low appendicular skeletal muscle mass by dual energy X-ray absorptiometry [DXA; M<7.0 kg/m2, F<5.4 kg/m2] or bioelectrical impedance analysis (BIA; M<7.0 kg/m2, F<5.7 kg/m2]; low muscle strength, defined as low grip strength [<28 kg for males, <18 kg for females]; or low physical performance, defined as <1.0 m/s in the 6 meter-walking test, ≤9 points in the Short Physical Performance Battery, or ≥12 seconds in the Five Times Sit-to-Stand test. Low bone mineral density is classified as osteopenia (between −1 SD and −2.5 SD T-score) and osteoporosis (< −2.5 SD T-score) according to the World Health Organization criteria

Patients with sarcopenia and low bone mineral density (osteopenia or osteoporosis) are classified as osteosarcopenia (Fig. 2) [13]. Participants who fulfill the following criteria will be considered eligible for the study: 1) postmenopausal females aged between 65 and 90 years; 2) diagnosed with SP according to the Asian Working Group of Sarcopenia [11] (low appendicular skeletal muscle mass by dual energy X-ray absorptiometry [DXA; M<7.0 kg/m^2^, F<5.4 kg/m^2^] or bioelectrical impedance analysis (BIA; M<7.0 kg/m^2^, F<5.7 kg/m^2^]; low muscle strength, defined as low grip strength [<28 kg for males, <18 kg for females]; or low physical performance, defined as <1.0 m/s in the 6 meter-walking test, ≤9 points in the Short Physical Performance Battery [SPPB], or ≥12 seconds in the Five Times Sit-to-Stand test); and 3) low bone mineral density diagnosed with osteopenia (T-score ≤-1.0 on DXA) or osteoporosis (T-score ≤-2.5 on DXA) [18].

#### Exclusion criteria

The exclusion criteria will be as follows: 1) secondary osteoporosis; 2) regularly taking medications that can affect the bone or muscle more than a month before the study (e.g., anticonvulsants, steroids); 3) a diagnosis of diabetes mellitus, thyroid disease, parathyroid disease, or Paget’s disease of bone; 4) active malignancy requiring treatment; 5) having implants, hardware, devices, or foreign material in the measurement area; 6) chronic kidney disease more severe than stage 3 (GFR<60 ml/min); 7) alcohol addiction; 8) a history of previous fragile fractures (radius, hip, or spine); 9) inability to understand or to participate in the study protocol; and 10) refusal to participate. In the case of patients affected by secondary osteoporosis, diabetes mellitus, thyroid disease, parathyroid disease, Paget’s disease of bone, or active malignancy, the authors exclude these individuals from the study due to the potential impact of these disorders on study results. The presence of implants, hardware, devices, or foreign material in the measurement area is considered inappropriate in this study due to the remodeling of adjacent tissues around the material in measuring the bone and body composition by DXA scan [19]. Chronic kidney disease more severe than stage 3 is regarded as a contraindication for a high-protein diet [20]. Furthermore, patients with alcohol addiction or a history of previous fragile fractures are also excluded from the study protocol due to the risk of falls or further fractures.

#### Exit criteria

The exit criteria will be as follows: 1) participants’ request, 2) serious side effects preventing further participation, and 3) not completing the entire study protocol.

### Randomization, allocation, and blinding

The study coordinator initially evaluated volunteered community-dwelling post-menopausal old female in Daejeon, Korea. After checking the inclusion criteria, eligible participants will be asked for consent to enroll in the study. After obtaining informed consent, all participants will be randomly assigned to two groups (1:1 allocation): the resistance exercise group and control group. Block randomization of four blocks will be used to balance allocation. After randomization, the study coordinator will handle participant allocation findings in a private manner. A single, trained physical therapist who is blinded to the participant groups will conduct each assessment for this study. Due to the nature of the study, participants allocated to the resistance exercise group will visit our gym twice per week, and will not be blinded. To ensure confidentiality and minimize potential bias, all participants will be instructed to avoid communication with any other researchers or physiotherapists regarding their allocated group.

### Intervention protocol

#### Exercise protocol

All participants will undergo interventions according to their allocated groups. Home exercise will be encouraged by our team by providing home training brochures (Supplemental Material 2) to the participants. Images from the home training brochures were copied with permission from www.physiotherapyexercise.com. Participants in the resistance exercise group will be instructed to visit the gym in our hospital twice per week for 24 weeks, and training logs containing prescribed exercises (number of sets, number of repetitions, and the required exercise intensity) will be provided. Circuit resistance training machines (Milon Industries GmbH, Emersacker, Germany) including eight strengthening exercises (4 for the lower extremities: leg press, leg abductor, leg flexor, leg extension; 3 for the upper extremities: chest press, row, dips; and 1 for core muscle exercise: back extension) will be used to train participants. Three phases of exercise will be applied to increase the intensity of the exercise. To determine the intensity of the exercise, we decided to apply the definition of the non-repetition maximum (nRM) and repetition maximum (RM), as in a previous report [21].

In phase 1 (1–8 weeks after allocation), each participant will be prescribed the range of repetitions, number of sets (1–2), movement velocity, and required intensity of the exercise. The required intensity of the exercise in phase 1 will be the nRM, defined as a “set endpoint when trainees complete a predetermined number of repetitions even though further repetitions could be completed” [21]. Therefore, participants allocated to the resistance exercise group will determine a weight on the machines for themselves which they can lift in the range of prescribed repetitions (8–15 times). During phase 1, participants will lift the self-determined weight 8–15 times (nRM: maximum effort minus 1–3 repetitions) in one or two sets, and in 2 seconds of concentric, 1 second of isometric, and 2 seconds of eccentric phase per repetition. All nine of the strengthening exercises previously described will be performed by the participants; 90–120 seconds of rest between the sets or exercises will be allowed.

In phase 2 (9–16 weeks after allocation), participants will be asked to increase the weight of the exercise until 7–10 reps are possible; the nRM will be applied to their exercise (maximum effort minus 1). Movement velocities will also be adjusted to 4 seconds of concentric, 1 second of isometric, and 4 seconds of eccentric phase per repetition. Otherwise, the same principles will be applied as in phase 1.

**Fig. 3:**
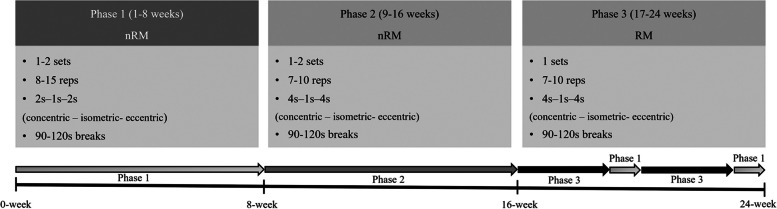
Protocol for exercise intervention, Reps, Repetitions; nRM, Nonrepetition Maximum; RM, Repetition Maximum

In phase 3, participants will be asked to apply the concept of the RM—defined as a “set endpoint when trainees complete the final repetition possible whereby if the next repetition was attempted, they would definitely achieve momentary failure” [21]—to each exercise. They will perform the number of repetitions judged to be possible in range of 7–10 reps. When the exercise intensity is increased to a high intensity, periodization is applied, including 3 weeks of high-intensity exercise (RM) and 1 week of moderate-intensity exercise (nRM, same as in phase 1) during the intervention period. In total, 60 minutes of exercise (10 minutes warm-up, 40 minutes main exercise, 10 minutes cool-down exercise) will be performed per session. Brief descriptions of the exercise protocols are depicted in Fig. 3.

Control groups will be provided with exercise brochures and encouraged to perform home exercise daily. The exercise brochures (Supplemental Material 2) will include stretching, balance training, back extensor strengthening, and other strengthening exercises. Performing home exercise at least twice per day will be instructed and encouraged.

### Nutritional supplementation

Basal nutritional surveys using 24-hour recall methods will be performed in both groups (exercise and control groups) by a trained dietician. Total calorie and protein intake will be assessed, and the Mini Nutritional Assessment-Short Form at T0 (0 weeks)—with the score ranging from 0–14 points—will be evaluated to assess the nutritional status of participants [22].

Protein supplementation during resistance exercises is known as significantly increasing the muscle size and strength in healthy adults [23]. In our study, two packs of protein complex powder (Selex core-protein; protein: 20 g [50% casein + 40% whey + 10% soy, total leucine: 3000 mg], vitamin D: 800 IU [20 µg], calcium: 300 mg, fat: 1.1 g, carbohydrate: 2.5 g; Maeil Dairies Co., Ltd., Seoul, South Korea) will be provided in daily basis; therefore, a total of 40 g of protein, 1600 IU of vitamin D, and 600 mg of calcium will be provided for the 24-week program in all participants, regardless of allocated group.

### Outcome measurement and participants’ timeline

**Fig. 4:**
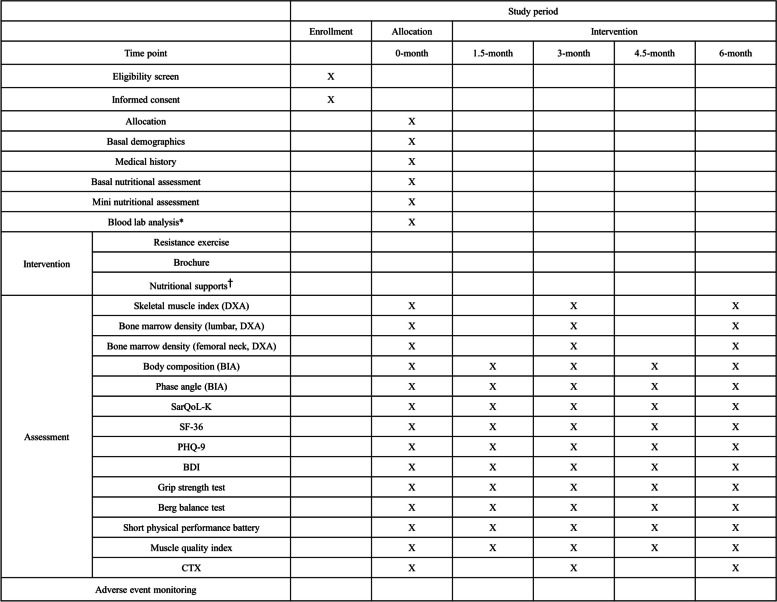
Patients' schedule of trial enrollment, interventions, and assessment. DXA, Dual X-ray Energy Absorptiometry; BIA, Bioelectrical Impedance Analysis; SarQoL-K, Korean version of the Sarcopenia Quality of Life; SF-36, 36-Item Short Form Survey; PHQ-9, Patients Health Questionnaire-9; BDI, Beck Depression Inventory, CTX: C-telopeptide (type 1 collagen). ^*^Blood lab analysis: Albumin, Pre-albumin, Aspartate Aminotransferase; AST, Alanine Aminotransferase; ALT, Alkaline Phosphatase; ALP calcium, Creatinine, Blood Urea Nitrogen; BUN, 25(OH) Vitamin D, Parathyroid hormone. ^†^Nutritional support: 2 packs of protein complex powder (Selex core-protein; protein: 20 g, vitamin D: 800 IU, calcium: 300 mg, fat: 1.1 g, carbohydrate: 2.5 g; Maeil Dairies Co., Ltd., Seoul, South Korea) will be provided

The primary outcomes of this study are changes in the skeletal muscle index (SMI) and BMD of the lumbar spine and total hip between the baseline and end of the intervention. The SMI is an indicator of skeletal muscle mass, which is calculated as the appendicular skeletal muscle mass divided by height squared (kg/m^2^). The secondary outcomes are quality of life measurement, functional assessment, bone turnover marker (C-telopeptide [type 1 collagen], CTX), BIA assessment (including body composition and whole-body phase angle), and muscle quality index (MQI). Participants will be screened for adverse events from study inclusion, to the end of the intervention. The schedule of the trial is illustrated in Fig. 4. After obtaining informed consent, basal measurements including demographic details (sex, age, height, weight, alcohol consumption, smoking history, and other medical history) will be surveyed by the research delegate. Weight (kg) divided by the height squared (cm^2^) will be used to compute the body mass index (BMI). Blood samples in fasting status will be collected from the antecubital veins of participants, and analyzed within 24 h of sampling. Albumin, pre-albumin, calcium, parathyroid hormone, serum vitamin D, creatinine levels will be evaluated at T0 (0 weeks).

### Primary outcome measurement

The primary outcomes of this trial will be changes in the SMI and BMD of the lumbar spine and total hip using DXA (Hologic, Marlborough, Massachusetts, USA) analysis from the baseline to end of intervention (24 weeks). Changes in body composition will be repeatedly measured at T0 (0 weeks), T2 (12 weeks), and T4 (24 weeks) to evaluate the effects of resistance training and nutritional support, as well as whether the effect gain by the intervention is maintained after training (Fig. 4).

### Secondary outcome measurement

#### Body composition and phase angle measurement by BIA

Body compositions, such as a lean body mass, body fat (%), and appendicular skeletal muscle mass, will be analyzed using multi-frequency BIA (S10; Inbody, Seoul, South Korea). The detailed protocol for BIA measurement follows the guidelines provided in the manufacturer's manual and is also informed by a previous study [24]. Before the BIA measurement, patients will be instructed not to eat, drink, or engage in resistance exercise for at least 2 hours. The BIA measurement will be conducted with patients in a supine position. The whole-body phase angle of the participants will be assessed by BIA four times every 6 weeks from the beginning to end of the intervention (Fig. 4) [25].

#### Quality of life and psychological assessment

The quality of life and depression status of participants will be measured using the Sarcopenia Quality of Life (SarQoL-K), 36-Item Short Form Survey, Patients Health Questionnaire-9 (PHQ-9), and Beck Depression Inventory (BDI).

The SarQoL-K questionnaire comprises twenty-two scaled questions and assesses the quality of life in community-dwelling elderly individuals aged 65 years and older who have sarcopenia. The questionnaire has been translated and validated in Korean [26] with scores ranging from 0 to 100.

The 36-Item Short Form Survey contains eight scaled scores and is a patient-reported survey regarding overall health, with scores ranging from 0–100; a higher score corresponds with a favorable health status [27].

The PHQ-9 and BDI are patient-reported questionnaires evaluating the depression status of participants. The PHQ-9 comprises nine items on a 4-point scale ranging from 0–3. A cutoff of 10 points is considered to indicate depression. The Korean PHQ-9 was validated in the older people [28], and will be used to assess the depression status of participants.

The BDI is one of the most widely used self-reported questionnaires for evaluating the severity of depression [29]. Twenty-one questions with multiple choices will be assessed and scored, with values of 0–3 for each question; the total score is therefore 0–63. Higher scores indicate more depressive symptoms in participants. The BDI has also been translated into a Korean version, and was proven to be reliable and valid for screening patients in older people [29].

#### Physical performance assessment

The physical performance of participants will be measured by handgrip strength (known to be a significant indicator of overall body strength, functional status, and even nutritional status [30–32]), the Berg Balance Test (BBT), and SPPB. Handgrip strength will be measured in the dominant hand using a handheld dynamometer (Jamar; Hoggan Scientific, Salt Lake City, Utah USA). All participants will be asked to sit in chair, adduct their shoulder, and flex their elbow 90°. They will then be asked to squeeze their hand to achieve the maximum effort isometric contraction; this will be repeated in triplicate, and the maximum reading of the gauze will be recorded, as previously recommended in Asian Working Group for Sarcopenia guidelines [11].

The BBT evaluates the functional balance of participants [33]. It evaluates both dynamic and static balance via 14 tasks. Each task is graded on a 5-point scale ranging from 0–4; the maximum score is 56 points, with a higher score indicating better balance and a lower chance of falls in the older people [34].

The SPPB is a widely used scale for evaluating the strength of the lower extremities, 4-m walking speed at usual pace, and static standing balance [35]. The SPPB is associated with falls reported in the older people, as well as mobility and disability [36]. The SPPB will be measured according to the standard previously reported [35]. Three tasks will be given to patients to complete in order to measure their standing balance, 4-m walking speed, and time to complete the Five Times Sit-to-Stand test. The SPPB's overall score scales from 0 to 12, with a higher score suggesting better participant performance.

#### Muscle quality index

The MQI is calculated from the common sit-to-stand test and reflects both the muscle power and informative functional index, incorporating the velocity of muscle shortening and anthropometric measures [37]. The MQI is calculated as follows [38]: MQI = ((leg length - 0.4) × body mass × gravity × 10) / time of sit-to-stand.

Using the taping method, the leg length is determined in this equation as the distance from the lateral malleolus to the greater trochanter of the femur. Body mass is measured in kilograms, the force of gravity is represented as 9.8 m/s^2^, and the time to complete the sit-to-stand test is the recorded time during the 10th repetition of sitting to standing in a standard chair from the sitting position. The MQI is validated and reliable [38], and reflects functional changes due to resistance exercise in the older people [37].

#### Bone turnover marker

We will measure bone turnover markers at three time points (T0, 0 weeks; T2, 12 weeks; T4, 24 weeks) during and immediately after the intervention to evaluate the effect of the intervention on bone metabolism. CTX is a known bone resorption marker, and higher levels are adversely associated with bone loss [39]. Fasting blood samples will be collected from the antecubital veins of participants, and CTX will be assayed via the Roche Cobas E801 electrochemiluminescence immunoassay (Roche Diagnostics, Basel, Switzerland) using the Elecsys beta-CrossLaps serum assay [40].

### Sample size calculation

The sample size was determined using SMI changes. A previous study demonstrated that the 28-week intervention with HI-RT and protein supplementation changed the SMI, with values of -0.03 ± 0.21 in the control group and 0.30 ± 0.22 in the intervention group [14]. A two-tailed, T-test-based sample test was performed according to the previous trial; the calculated sample size of 13 participants per group corresponds with a 95% power (1-β), type-I alpha error =0.05, and effect size of d=1.53. We decided to include at least 17 people each group, assuming a 25% dropout rate.

### Data management

Our research team will collect and process all data from participants, which will be stored on a platform accessible to the research team alone. All obtained information will be retained for 5 years. The backup database will be regularly updated. The anonymized dataset will be made available upon request to the corresponding author.

### Statistical analysis

All analyses will be carried out using IBM SPSS Statistics (version 23.0; IBM, Armonk, New York, USA), with a significance level of 5% and a 95% CI. Descriptive statistics will be applied to present the subject demographics. Intention-to-treat analysis will be performed to analyze the results, and the independent T-test will be used to compare the baseline values between groups. For the analysis of primary outcomes, independent t-test or Mann-Whitney U test for comparisons between changes from the baseline to the end of the intervention will be applied according to the distribution of data by Shapiro-Wilk test. The data will be described using means, standard deviations, and 95% confidence intervals (CIs). The method for handling missing data will be chosen after the study is finished based on the distribution of the data. A mixed-effects model or generalized estimating equations will be employed using one between-subject factor (group: intervention and control) and one within-subject factor to compare the secondary outcomes and find differences in outcomes between groups with time.

### Predicted adverse events and monitoring

The potential for adverse events and risks, as reviewed by the institutional review board and primary investigator, will be minimal. The potential adverse events that could occur in the exercise group during the intervention will be observed by therapists include fatigue, arthralgia, exercise-induced injuries, or falls; participants in the control group will not be queried regarding adverse events. Additionally, the therapists will report the primary research coordinator and handle the problem as quickly as feasible if the patient's discomfort during exercise worsens or if the symptoms will not go away before the next exercise session. The number and seriousness of the adverse events will be reported.

### Ethics and dissemination

The institutional review board of our hospital approved all study procedures, and the study was registered with Clinical Research Information Service of Republic of Korea (KCT0008291). The trial participants and institutional review board will be notified if significant changes are made to the study protocol. During the study, participants’ personal information will only be accessible by the qualified investigators. The trial results will be published in the journal, and the report of results will be posted on the funding institute’s site (accessible to the public, participants, and healthcare professionals).

## Discussion

In this research proposal, we describe a single-center, randomized clinical trial comparing resistance exercise plus nutritional support in patients with OS in Daejeon, Korea. In this trial, we aim to determine the effects of resistance exercise and nutritional support on the SMI and BMD of postmenopausal females with OS. Functional assessment (including the grab strength test, BBT, and SPPB) and micro-architectural assessment of bone and muscle (including the CTX and whole-body phase angle) will be included in this trial, making the conclusion from this trial more clearly.

Research regarding the non-pharmacological management of OS has been published in the past. The FrOST study evaluated the effects of HI-RT and protein supplementation for about 18 months; they observed significant positive effects on the SP Z-score, and BMD at the lumbar spine and total hip [15]. However, the inclusion of only males, and differences in protein intake between the intervention and control groups, were the limitations of the study. Banitalebi et al. [41] reported the effect of elastic band resistance training in female patients with osteosarcopenic obesity. They included 63 females, and performed 12 weeks of elastic band resistance training, resulting in improvements in the osteosarcopenic obesity Z-score, 30-s chair raise test, and hand grip strength test; no improvements were observed in CTX, gait speed, and BMD. However, the intensity of resistance training was only determined by the resistance of the elastic band, and the relatively short training duration may have been an obstacle against proving the conclusion of the trial.

In this trial, we will attempt to prove the long-term effects of resistance training (24 weeks) and nutritional support (protein, vitamin D, and calcium) in females with OS. HI-RT will be applied, and the concept of the nRM/RM will be applied during the intervention [21]. Additionally, the same amount of protein, vitamin D, and calcium will be prescribed in both the intervention and control groups.

Nowadays, micro-architectural assessment of bone and muscle metabolism is commonly used in clinical practice. For example, bone turn over markers such as osteocalcin, CTX, and bone-specific alkaline phosphatase are used to monitor the effect of management in osteoporosis [42]. Fathi et al. also revealed that CTX was associated with OS in the multivariable logistic regression model (OR=4.363, 95% CI: 1.389–15.474). Likewise, phase angle analysis using BIA reflects muscle quality, and is used to detect SP [25]; additionally, these values could reflect the effect of resistance training in older patients [43]. Therefore, utilizing these assessments to evaluate the micro-architectural status of bone and muscle in participants could demonstrate the more explicit effects of the intervention.

The psychological aspects of patients with OS have not been well established. Huo et al. [6] performed a cross-sectional study of 679 participants and concluded that patients with OS exhibited more depressive symptoms than other comparative groups. However, to our knowledge, assessment of the psychological status and management of depressive symptoms in patients with OS have not been studied. Resistance exercise is well known to significantly reduce depressive symptoms among adults [44]. Therefore, in this trial, we could evaluate the psychological assessment and improvement due to the intervention, with the aim of eventually using the data to improve the non-pharmacological management and provide more clinical benefits to patients with OS.

There are several limitations to the protocol of this randomized trial. Firstly, we only included post-menopausal women residing in the community who volunteered as potential participants. This approach might be perceived as convenience sampling, despite the subsequent proper group randomization following informed consent. This introduces a potential source of bias in the study. Secondly, the sample size for this randomized trial is around 34 post-menopausal patients with osteosarcopenia. Despite the calculated estimated sample size from previous reports [14], the small sample size could reduce the statistical power.

In conclusion, this randomized trial will demonstrate the benefits of resistance exercise and nutritional support on bone and muscle health in female patients with OS, and provide long-term follow-up data.

## Trial status

Protocol version number V1.0, 07 March 2023. The institutional review board of our hospital approved all study procedures, and the study was registered with Clinical Research Information Service of Republic of Korea (KCT0008291). Patient recruitment began in March 2023, and the completion date for recruitment is in November 2023. The experiments are currently in progress, with the estimated date of completion set for December 2024.

## Supplementary Information

**Additional file 1.** SPIRIT 2013 Checklist: Recommended items to address in a clinical trial protocol and related documents*.

**Additional file 2: Supplemental material 2.** Exercise for the patients with osteosarcopenia.

## Data Availability

Not applicable.

## Abbreviations

BBT: Berg Balance Test
BDI: Beck Depression Inventory
BIA: Bioelectrical Impedance Analysis
BMD: Bone Marrow Density
BMI: Body Mass Index
CTX: C-telopeptide (type 1 collagen)
DXA: Dual-energy X-ray Absorptiometry
HI-RT: High-intensity Resistance Exercise
MQI: Muscle Quality Index
nRM: Non-repetition Maximum
OS: Osteosarcopenia
PHQ-9: Patients Health Questionnaire-9
RCT: Randomized Controlled Trial
RM: Repetition Maximum
SMD: Standardized Mean Difference
SMI: Skeletal Muscle Index
SP: Sarcopenia
SPPB: Short Physical Performance Battery
