# Supplementary material for: Effects of resistance training and nutritional support on osteosarcopenia in older, community-dwelling postmenopausal Korean females (ERTO-K study): a study protocol
Source: BMC Geriatr. 2024 Jan 17;24:68. doi: 10.1186/s12877-024-04667-1 (PMC10792803; doi:10.1186/s12877-024-04667-1)
Supplement: Supplementary file 2 — Additional file 2: Supplemental material 2. Exercise for the patients with osteosarcopenia. [file 12877_2024_4667_MOESM2_ESM.docx]

**Supplemental material 2. Exercise for the patients with osteosarcopenia**

**Stretching exercises (8 exercises)**

| Exercises | Instructions |
| --- | --- |
| Active assisted shoulder range of motion exercise 1 | position yourself sitting in a chair with your hands clasped together on your lap. Raise your hands upwards to rest on the top of your head. Slide your hands behind your head as far as you can. Push your elbows back as you can. Return your hand to your lab. |
| Active assisted shoulder range of motion exercise 2 | Position yourself sitting in a chair with your hands clasped together on your lap. Raise your hands upwards to rest on the top of your head. Slide your hands behind your head as far as you can. Reach upwards and slightly backwards with both hands. Straighten their elbows and hold the position for 5 seconds. Return your hands to your lap. |
| Posterior capsule stretch in standing | Position yourself in standing. Use one hand to stretch your opposite shoulder by pulling your arm across your body. |
| Lumbar extension in standing | Position yourself in standing with your feet apart and hands on your lower back. Support your lower back with your hands while bending back as far as is comfortable. Ensure you keep your knees straight. |
| Wrist and finger extensor stretch in standing | Position yourself in standing with your arm outstretched and your palm facing down. Bend your fingers and your wrist with your other hand. Ensure that your elbow remains straight. |
| Wrist and finger flexor stretch in standing | Position yourself in standing with your arm outstretched and your palm facing upwards. Straighten your fingers and bend your wrist back with your other hand. Ensure that your elbow remains straight. |
| Quadriceps stretch and hip stretch in standing | Position yourself in standing with one knee bent. Hold onto the ankle of your bent knee and pull it towards your bottom. Ensure to keep your knee under your hip and your shoulders over the hips. |
| Gastrocnemius stretch in standing | Position yourself standing with one leg in front of the other and your hands resting on a wall. Lunge forwards while keeping your back leg straight. Ensure that both feet point forwards and your back heel remains on the ground. |

**Spine extension exercises (2 exercises)**

| Exercises | Instructions |
| --- | --- |
| Shoulder retractor strengthening in standing | Position yourself standing up straight. Practice pulling your shoulder blades together. |
| Hip extension in standing against a wall | Position yourself standing with your shoulders, bottom and ankles against a wall. Practice moving your bottom away from the wall. Ensure that your knees remain straight and your shoulders and feet remain against the wall. |

**Strengthening exercises (8 exercises)**

| Exercises | Instructions |
| --- | --- |
| Straight leg raise | Position yourself lying on your back with your legs straight. Start with your leg straight and your heel on the bed. Finish with your leg straight and your heel above the bed. |
| Bridging to end of range | Position yourself lying on your back with your knees bent. Tighten the muscles in your bottom and lift your bottom off the bed. |
| Hip abduction in side lying | Position yourself lying on your side. Start with your top leg resting on the bed. Lift your leg away from the bed leading with your heel. Finish with your leg away from the bed. Ensure that your hip and knee are kept straight and your foot points forwards. |
| Hip extensor strengthening in prone without weights | Position yourself lying on your stomach. Start with your leg on the bed. Finish with your leg off the bed. |
| Wall squatting | Position yourself leaning with your back against a wall, your feet about 20 cm from the wall and arms crossed. Start with your knees straight. Finish with your knees bent. Ensure that your knees stay in line with your feet and your weight is equally borne through both legs. |
| Bilateral calf raises | Position yourself standing with your feet together. Start with your heels on the ground. Finish with your heels off the ground. |
| Wall push ups | Position yourself standing and leaning forward with hands supported on a wall. Practice doing push-ups through your hands so that your chest lifts away from the wall. Ensure to keep your hips straight. |
| Elbow flexor strengthening in supine using free weights | Position yourself lying on your back. Start with your arm down beside your body. Finish with your hand up near your shoulder. Ensure that your elbow is held beside your body. |

**Balancing exercises (8 exercises)**

| Exercises | Instructions |
| --- | --- |
| Transferring weight laterally in standing | Position yourself standing with your feet slightly apart. Practice transferring your weight from one leg to the other. Ensure that your shoulders remain over your hips. |
| Stepping sideways | Position yourself standing with your feet together. Practice stepping sideways. Ensure that your knees are kept straight and your feet point forwards. |
| Cross over sidestep | Position yourself standing with your feet shoulder width apart. Practice walking sideways by taking a big step to the side crossing your trailing leg in front of your leading leg. Ensure that you walk sideways rather than diagonally. |
| Stand and look behind | Position yourself standing with your feet slightly apart. Practice turning your head to look over your shoulder. Aim to look around behind you as far as you can, without moving your feet or taking a step. |
| Turning around on the spot | Position yourself standing up. Practice turning around on the spot. |
| Standing and bending and straightening the knees quickly | Position yourself standing with a table nearby for support. Practice standing on your affected leg and rapidly bending and straightening your knee through a small range. Ensure that your affected knee does not lock back past straight and hand support is used only if necessary. |
| Stand and pivot on one leg | Position yourself in standing with a stable support nearby. Practice standing on one leg and pivoting (turn foot and body) from side to side, keeping your weight on the ball of the pivot foot as you turn. Ensure that hand support is used only if you feel unsteady. |
| Walking in a figure-of-eight | Position yourself standing up. Practice walking in a figure-of-eight pattern. |

Acknowledgement: These instruction and images used in the booklets are copied with permission from www.physiotherapyexercises.com: freely availably exercise prescribing software
